# Quality improvement project to routinely identify sensory and eating challenges in childhood neurodevelopmental movement disorders

**DOI:** 10.1136/bmjoq-2024-002919

**Published:** 2025-07-17

**Authors:** Sandra-Eve Bamigbade, Sara Sopena, Tammy Hedderly, Osman Malik, Tamsin Owen, Amanda K Ludlow

**Affiliations:** 1 University of Hertfordshire Department of Psychology Sport and Geography, Hatfield, UK; 2 University of Hertfordshire Centre for Research in Public Health and Community Care, Hatfield, UK; 3 Tics and Neurodevelopmental Movements Team (TANDeM), Children’s Neurosciences, Evelina London Children’s Hospital, Guy's and St Thomas’ NHS Foundation Trust, London, UK; 4 Department of Women and Children’s Health, School of Life Course Sciences (SoLCS), King’s College London, London, UK

**Keywords:** Quality improvement, Paediatrics, PDSA

## Abstract

Sensory, eating and mealtime (SEM) challenges are common among young people with neurodevelopmental movement disorders but are rarely assessed during initial clinical consultations. This quality improvement project aimed to evaluate the impact of routine SEM screening on identifying these challenges and improving documentation and follow-up in a specialist paediatric movement disorder clinic in England. Using the SHIFT-Evidence Framework and a Plan-Do-Study-Act approach, the project implemented and evaluated a series of interventions. In the **‘PLAN’** phase, clinicians participated in a workshop to raise awareness of SEM challenges and inform the development of screening questions to support routine SEM assessment. The **‘DO’** phase involved implementing routine SEM screening during a 3 month trial, supported by active measures such as project champions, weekly reminders and team discussions to encourage sustained practice. The **‘STUDY’** phase included analysis of assessment outcome letters from three time points (baseline, trial and outcome retention phases) to evaluate changes in documentation and the sustainability of improvements. In the **‘ACT’** phase, findings were shared with the team, resulting in improved signposting, targeted recommendations and ongoing collaborations with feeding clinics. Findings demonstrated increased documentation of SEM challenges in assessment letters, with mentions rising from 33% at baseline to 71.9% during the trial and 64.3% in the retention phase. However, actionable recommendations and interventions remained limited during the trial but showed improvement in the retention phase, where letters included more tailored guidance and specific advice for SEM challenges. This project highlights the prevalence of SEM challenges among young people with neurodevelopmental movement disorders and underscores the importance of routine SEM screening. Developing standardised assessment tools and protocols could further aid clinicians in identifying and addressing these challenges during initial assessments.

**WHAT IS ALREADY KNOWN ON THIS TOPIC**

- Emerging research is beginning to highlight the presence of sensory, eating and mealtime (SEM) difficulties in people with neurodevelopmental movement disorders. However, this research has yet to clearly influence clinical practice.

**WHAT THIS STUDY ADDS**

- This project demonstrates how routine assessment of SEM challenges in paediatric movement disorder populations creates opportunities to identify unmet needs and deliver targeted support, ultimately improving quality of life. It also shows how such practices can be sustained beyond the active intervention phase.

**HOW THIS STUDY MIGHT AFFECT RESEARCH, PRACTICE OR POLICY**

- High-quality assessments are essential for integrating routine SEM screening into evaluations of individuals with neurodevelopmental disorders. This study outlines practical steps for embedding SEM assessment into service infrastructure, including standardised forms, checklists and staff training, and supported by quality assurance processes such as audit and supervision. We recommend developing accessible tools to streamline the identification of current challenges, supporting the creation of comprehensive management protocols for SEM issues identified during assessments.

## Problem

 Children with neurodevelopmental movement disorders such as tic disorders appear to face complex combinations of nutritional, feeding skill and psychosocial challenges related to feeding and mealtimes.^1 2^ Common terms like ‘food refusal’ and ‘food selectivity’ can understate the significance of eating concerns in this population. For example, children with tic disorders are significantly less likely to meet daily reference nutrient intake guidelines for various vitamins compared with their peers without such disorders.^3 4^

Participation in daily activities, such as eating, is critical for overall health and quality of life. Interventions that include comprehensive assessments to address eating-related challenges have shown positive outcomes in children with autism.^5^ Similarly, recognising and addressing eating concerns in children with neurodevelopmental movement disorders may help reduce nutritional risks, support caregivers and enhance participation in meaningful daily activities.

## Background

Tics and stereotypies emerge as prevalent forms of repetitive and intricate motor behaviours observed throughout neurodevelopment. Tics are sudden, repetitive movements or sounds. While tics may manifest temporarily during childhood without raising pathological concerns, their persistence can lead to distress, social limitations or, in severe cases, potential physical risks such as malignant tics.^6^ Unlike tics, stereotypies cluster and tend to involve the same muscle groups in a consistent, repetitive and rhythmic pattern.^7^

When tics are severe and/or frequently occur, they are often associated with having a tic disorder. Tic disorders are characterised by involuntary, repetitive and non-rhythmic motor and phonic tics. For a diagnosis of Tourette syndrome, both types of tics must be present, and they must have been present for at least 1 year since childhood.^8^ If only motor or phonic tics are present, a diagnosis of motor/vocal persistent tic disorder may be assigned. Tics present for under a year may be classified as provisional tic disorder,^8^ while functional tic-like behaviours are categorised separately.^7^

Eating behaviours and mealtimes pose considerable concerns for young people with tic disorders and their families.^9^ For example, both mothers and adolescents with tic disorders have reported food-related challenges, such as difficulty staying seated during meals, avoiding eating altogether and experiencing stress due to heightened attention drawn to their tics during mealtimes.^1 10 11^ Notably, recent research indicates heightened levels of food selectivity (aversion to familiar and unfamiliar foods based on certain tastes, textures or colour), food neophobia (unwillingness to try novel foods) and avoidant/restrictive eating behaviours in individuals with tic disorders when compared with typically developing peers irrespective of age.^12 13^

While selective eating is observed across neurodevelopmental disorders (NDDs),^14^ it is most commonly associated with autism, with prevalence rates ranging between 45% and 90% (for review, see^15^). Notably, increased sensory sensitivity and rigidity have been identified as key transdiagnostic mechanisms in the development and persistence of selective eating, observed in both neurotypical and neurodevelopmental populations,^16^,^18^ highlighting the vulnerability of individuals with NDDs to selective eating and associated mealtime challenges.^1 19^ Consequently, the focus on autism in selective eating research may inadvertently contribute to the underassessment of sensory and selective eating challenges among individuals with other NDDs, such as Tourette syndrome and other neurodevelopmental movement disorders.^6^

It is unclear whether specialised paediatric movement disorder services effectively address sensory, eating and mealtime (SEM) challenges in young people with persistent neurodevelopmental movement disorders. Families do not typically raise these issues spontaneously, requiring professionals to actively inquire about them.^20^ This project aimed to determine whether incorporating SEM questions into routine assessments would increase the number of individuals identified with SEM challenges during initial assessments. Additionally, we explored whether SEM screening would continue outside the trial period without active monitoring and whether targeted support would be developed and offered as a result. By addressing SEM challenges early, clinicians could help mitigate their impact on participation in daily activities such as eating and improve the overall quality of life for young people with neurodevelopmental movement disorders.

## Methods

### Setting

This quality improvement (QI) project was conducted within a tertiary multidisciplinary paediatric movement disorder service in England and was approved by the associated NHS trust (project: 14953). Research ethics approval was not applicable as this project was classified as QI, focusing on enhancing service delivery within a specific setting and improving internal processes within the organisation. The project involved minimal risk to patients, as it evaluated modifications to existing clinical practices within the NHS-approved framework. Our approach was guided by the SHIFT-Evidence Framework,^21^ which serves as a theoretical framework outlining 12 actionable ‘simple rules’. For example, in accordance with the principles outlined in the SHIFT-Evidence Framework, through consultations with clinicians and observation of routine tic assessments, we aimed to understand assessment practices and whether SEM experiences were addressed during initial assessments. We identified that SEM experiences were not routinely assessed and thus explored barriers to this and developed solutions to improve routine assessment. Finally, we assessed whether improvement was achieved and to capture and share learning through analysis of outcome letters over three different time periods (baseline, trial at 3 months after baseline and outcome retention at 1 year after baseline). We prioritised formal education of staff members, regular discussions at team meetings and email reminders as integral components of our project.^22^ Moreover, throughout the project, the team actively engaged in discussions to develop new improvement strategies and coordinate data collection and analysis.

**Figure 1:**
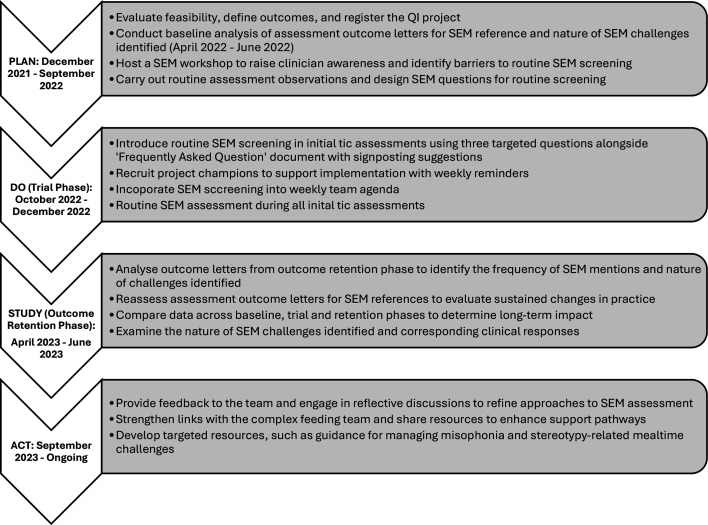
Processes involved in the PDSA cycle. PDSA, Plan-Do-Study-Act; QI, quality improvement; SEM, sensory, eating and mealtime.

Method-wise, a Plan-Do-Study-Act (PDSA) approach was adopted.^23^ The PDSA entails iterative phases of planning interventions (Plan), implementing these interventions on a small scale (Do), studying the outcomes (Study) and acting on insights gained to refine interventions and continue the improvement process (Act). The process is illustrated in figure 1.

## Plan

The planning phase was the most labour-intensive for the core project team, involving extensive discussions about the purpose and role of the organisation, as well as the practicalities of potentially increasing the burden on children and families during initial assessments. Key decisions were made regarding the outcome measures, monitoring processes, clinician roles in the intervention, communication strategies with the wider team, staff training, start dates, stages of the improvement cycles and scheduling progress review meetings.

To establish a baseline, assessment outcome letters from April 2022 to June 2022 were reviewed to identify references to SEM experiences. Since patients are typically evaluated only once, within-case letter comparisons were not feasible; instead, case comparisons were conducted to assess shifts in clinical practices. Qualitative data from the letters were categorised as either ‘mentioned’ or ‘not mentioned’, enabling an evaluation of whether future initiatives would lead to increased SEM documentation. These findings were presented during a training workshop for clinicians to highlight the baseline level of SEM documentation and raise awareness.

The workshop, co-presented by the lead author, focused on enhancing clinicians’ understanding of SEM challenges experienced by young people with NDDs, particularly tic disorders, as evidenced by research findings. Following the presentation, a brief reflective practice session was conducted to discuss the implications of the findings and prior clinical experiences with SEM challenges. Insights from this session, combined with clinic observations by the lead author, informed the development of three SEM screening questions. These questions were designed to address sensory sensitivities, selective eating and mealtime challenges, laying the foundation for the trial phase in October–December 2022.

## Do

The trial phase began on 1 October 2022 and continued until the end of December 2022, shortly after the workshop for clinicians. The main objective was to streamline the process of routinely assessing and identifying SEM challenges during initial assessments. The clinicians’ role was to ensure the three targeted screening questions were consistently asked and documented in all outcome letters.

The three questions, developed as research priorities based on existing literature and practices in other NDD services (eg, autism), were designed to be brief and practical for inclusion in initial assessments. These questions addressed:

**Sensory sensitivity:** ‘*Would you describe yourself/your child as someone who is sensitive to tastes, smells, textures and/or sounds?’*

**Selective eating:** ‘*Would you describe yourself/your child as a picky/fussy eater?’*

**Mealtime challenges:** ‘*Some people report that their tics can be problematic during mealtimes inside and outside of the family home. Is this the case for you/your child?’*

Clinicians were encouraged to adapt these questions to suit their assessment style while maintaining a focus on assessing SEM experiences. If service users responded ‘yes’ to any of the questions, follow-up prompts were advised to ascertain the nature and impact of challenges. The questions were designed to be concise to minimise additional burden on staff and to avoid compromising other assessment duties.

To support implementation, clinicians were provided with a ‘Frequently Asked Questions’ document, which included project details and a curated list of resources for addressing sensory needs and eating difficulties. This ensured easy access to information and practical tools to assist with SEM challenges.

Additionally, project champions were recruited to maintain team engagement with the initiative. Their responsibilities included incorporating project-related discussions into weekly team meetings and sending regular reminders to reinforce the importance of routine SEM assessments.

## Study

During the trial, active efforts by project champions and the service aimed to encourage routine SEM screening in all initial assessments. To evaluate the impact of these efforts, outcome letters were analysed across three time periods—baseline (April–June 2022), trial (October–December 2022) and retention (April–June 2023).

A comparison was made between the proportion of letters mentioning SEM experiences across the three time intervals to measure the effectiveness of the interventions during the trial and whether improvements were sustained in the retention phase. X^2^ test of independence was used to determine whether the differences in SEM documentation across these intervals were statistically significant. Where letters mentioned SEM experiences, further analysis was conducted to determine:

1. Whether a specific SEM challenge, difficulty or unmet need was identified.
2. The type of challenge or need documented (eg, sensory sensitivity, selective eating).
3. The clinical response provided (eg, signposting, advice, referral, psychoeducation).

This approach allowed the team to assess both the short-term impact of trial interventions and the longer-term sustainability of routine SEM screening during the retention phase while capturing differences across all three intervals.

## Results

A total of 81 assessment outcome letters were included in this QI project. Patients who were assessed during the baseline, trial and outcome retention formed different population groups and were between four and 17 years of age, almost equally split between male and female, and were primarily diagnosed with either Tourette syndrome, followed by functional tic-like behaviours, see table 1.

**Table 1:** Patient characteristics

	Baseline(n=21)	Trial(n=32)	Outcome retention(n=28)	All data(n=81)
Age				
Mean (SD)	11.57 (2.66)	12.14 (3.25)	9.71 (2.96)	11.15 (3.18%)
Range (years)	7–16	5–17	4–14	4–17
Gender				
Male (%)	9 (43%)	16 (50%)	15 (54%)	55 (45%)
Female (%)	11 (52%)	15 (47%)	13 (46%)	53 (43%)
Trans (%)	0 (0%)	1 (3%)	0 (0%)	1 (1%)
Non-binary (%)	1 (5%)	0 (0%)	0 (0%)	1 (1%)
Primary neurodevelopmental movement disorder diagnosis				
Tourette syndrome	7 (33%)	19 (59%)	18 (64%)	44 (54%)
Functional tic disorder	8 (38%)	11 (34%)	3 (11%)	22 (27%)
Stereotypic movement disorder	5 (24%)	1 (3%)	5 (18%)	11 (14%)
Other tic disorders	1 (5%)	1 (3%)	2 (7%)	4 (5%)

To measure the effectiveness of our efforts to increase routine assessment both while active measures were in place to support routine assessment (trial) and after (outcome retention), a comparison was made between the proportion of letters that mentioned SEM, in baseline, trial and outcome retention. X^2^ test of independence showed that there was a significant association between sensory challenges and needs documented in the outcome assessment across three time intervals, *X*^2^ (3, n=81) = 8.25, p=0.02.

### Sensory-related eating behaviours and mealtime experiences

When eating and mealtime experiences were documented, they predominantly related to sensory processing challenges, such as aversions to specific smells or the sound of others eating. Sensory-based eating challenges were documented in 17.3% of letters overall, with the highest proportion noted during the trial phase (34.4%) and fewer mentions in the baseline (9.5%) and retention (3.6%) phases. The severity of sensory-related impacts on dietary range varied, with some cases involving mild aversions and others severe enough to result in weight loss and nutritional deficiencies. Notably, only one child was already under the care of a dietician, while two other children experiencing significant weight loss and food intake limitations were not referred for specialist support, despite clinical need.

### Rigidity or compulsion-related eating behaviours and mealtime experiences

Eating behaviours influenced by rigidity or compulsions were less commonly documented, appearing in 6.2% of letters overall. Examples included inflexible food rules (n=4), food neophobia (fear of unfamiliar foods, n=3) and engagement in ritualistic eating behaviours (n=1). Mealtime rigidity, documented in only 2.5% of letters, included strict utensil and cookware preferences (n=1) and concerns about food sharing and contamination (n=1). These behaviours were mentioned in the baseline and trial phases but not observed in the retention phase.

### Movement-related eating and mealtime difficulties

Movement-related difficulties, such as those arising from tics, stereotypies and intense imagery movements (IIM), were documented in 4.9% of letters overall. These included oral tics that disrupted eating, one instance of choking and a case of problematic hand tics. IIMs were specifically noted during the retention phase, highlighting their potential impact on eating and mealtime experiences. Movement-related challenges were most frequently mentioned during the trial phase (9.4%) but declined in the retention phase (3.6%).

### Interventions for SEM challenges

Despite the identification of sensory, rigidity and movement-related eating and mealtime challenges, only 11.1% of letters that identified eating and mealtime challenges also documented advice or signposted for support. During the trial phase, documented interventions included sensory strategies to aid regulation (eg, in school settings) and general signposting to autism resources. However, these recommendations were often generic and lacked specific, actionable guidance tailored to individual needs.

In the retention phase, documented advice became more targeted. For example, one letter provided strategies to address sensory and mealtime difficulties linked to ADHD medication side effects (appetite suppression) and sensory overwhelm in the school canteen. This letter offered specific recommendations for managing these dual challenges. Despite this, many cases would have benefited from comprehensive formulations of SEM challenges and personalised recommendations, highlighting a need for improved clinical practice.

Over the 15 month study period (April 2022 to June 2023), there were more eating and mealtime challenges identified in 33% of assessment outcome letters, with surprisingly more identified during baseline (62%) compared with the trial (22%) and retention outcome phases (25%) (see table 2). However, X^2^ test of independence showed no statistically significant association between the number of mentions and the assessment phase (*X²*(3, n=81) = 10.47, p<0.01. There were similar levels of eating and mealtime advice and/or signposting across the three time periods (baseline 29%; trial 1%; outcome retention phrase 11%).

**Table 2:** Sensory, eating and mealtime challenges documented in assessment outcome letters

	Baselineletters(n=21)	Trial(n=32)	Outcome retention(n=28)	All letters(n=81)
SEM challenges/needs	7 (33%)	23 (72%)	18 (64%)	59 (73%)
Eating or mealtime difficulties	13 (62%)	7 (22%)	7 (25%)	27 (33)
Named or described sensory-based eating challenges	2 (10%)	11 (34%)	1 (4%)	14 (17%)
Named or described sensory-based mealtime challenges	0 (0%)	3 (9%)	3 (11%)	6 (7%)
Named or described rigidity/compulsion-based eating behaviours	2 (10%)	3 (9%)	0 (0%)	5 (6%)
Named or described rigidity/compulsion-based mealtime behaviours	2 (10%)	0 (0%)	0 (0%)	2 (3%)
Named or described movement-related eating difficulties	0 (0%)	3 (9%)	1 (4%)	4 (5%)
Named or described movement-related mealtime challenges	1 (6%)	3 (10%)	1 (4%)	5 (6%)
Eating or mealtime advice or signposting documented	0 (0%)	2 (29%)	1 (14%)	3 (11%)

SEM, sensory, eating and mealtime.

Sensory processing differences were also found to be a common experience among children with paediatric neurodevelopmental movement disorders. Over the 15 month study period (April 2022 to June 2023), sensory differences were identified in 53% of assessment outcome letters, with a marked increase in documentation during the retention (64%) and trial (56%) phases compared with baseline (33%) (see table 3). However, X^2^ test of independence showed no statistically significant association between the number of mentions and the assessment phase (*X²*(3, n=81) = 4.83, p=0.09).

**Table 3:** Sensory experiences documented in assessment outcome letters

	Baselinen=21	Trialn=32	Outcome retentionn=28	All lettersn=81
Sensory processing differences	7 (33%)	18 (56%)	18 (64%)	43 (53%)
Named or described sensory sensitivities	5 (24%)	17 (53%)	12 (43%)	34 (42%)
Named or described sensory seeking behaviours	1 (5%)	1 (3%)	8 (29%)	10 (12%)
Misophonia (aversion to specific sounds)	2 (10%)	2 (6%)	2 (7%)	6 (7%)
Misokinesia (aversion to specific movements)	1 (5%)	1 (3%)	0 (0%)	2 (3%)
Sensory advice or signposting documented	0 (0%)	4 (7.1%)	6 (33.3%)	10 (23.3%)

Of the sensory experiences documented, sensory sensitivities were the most frequently mentioned, appearing in 42% of letters overall. These sensitivities are commonly related to clothing (eg, discomfort with socks, shoes, tags and labels) and environmental triggers (eg, noise, crowds).

### Sensory advice or signposting

Despite the prevalence of sensory processing differences, only 23.3% of letters that mentioned sensory challenges also documented advice or signposting to manage these needs. This was most evident in the retention phase, where 33.3% of letters included advice or recommendations, compared with just 7.1% during the trial phase and none at baseline. The advice provided often focused on school-based accommodations, potentially to support requests for adjustments or validate the need for interventions in educational settings.

In many cases, sensory needs were described as mild or already well-managed by families (eg, through strategies such as noise-cancelling headphones or fidget toys), potentially explaining the lack of recommendations in some letters. However, the limited documentation of advice in letters highlights a missed opportunity to provide tailored guidance or referrals to resources, particularly for those with more significant challenges.

## Act

The findings from the study were shared with the clinical team, prompting discussions about service adaptations to address identified gaps and improve SEM screening and management. Several changes were agreed on to enhance practice and ensure better support for children and families. Table 4 provides an overview of the key findings from the retention phase and the corresponding actions taken or planned by the service.

**Table 4:** Reflective practice actions taken (ACT phase)

Findings	Responses and next steps
SEM challenges continued to be identified in outcome letters during the retention phase, though fewer were detailed.	Provided feedback to the clinical team, emphasising the importance of consistent documentation and detailed descriptions of SEM challenges in outcome letters.
Sensory-based eating challenges and misophonia during mealtimes were common, but actionable recommendations were still limited.	Developed targeted resources, including a leaflet addressing misophonia and improved guidance for managing sensory-based eating challenges.
Clinicians expressed uncertainty about referral thresholds for feeding services.	Strengthened communication with the specialist feeding team to clarify referral criteria, designated a contact person for consultations and improved guidance on addressing challenges below referral thresholds.
Documentation of SEM challenges increased, but psychoeducation and specific guidance remained inconsistent.	Encouraged clinicians to include tailored recommendations in outcome letters (eg, school adjustments, home strategies) and began developing resources with psychoeducation and generic advice.
Time constraints and competing priorities remained barriers to sustained improvements in SEM screening and management.	Explored the development of brief, standardised screening tools (eg, self-report questionnaires) to streamline SEM identification and reduce clinician workload.
Challenges raised in letters were inconsistently linked to appropriate follow-up or interventions.	Proposed consistent follow-up documentation in letters, including formulations to better inform families and future clinicians about SEM-related needs and responses.

SEM, sensory, eating and mealtime.

## Discussion

This QI project aimed to address the underassessment of SEM challenges among children with neurodevelopmental movement disorders. During a 3 month trial, we evaluated assessment outcome letters to determine whether introducing SEM screening increased the number of letters mentioning these experiences. To assess whether our awareness-raising measures had a lasting impact on clinical practice, we analysed letters a year after the baseline period, which was 3 months after the trial ended. This approach allowed us to see whether the identification of SEM increased during the active intervention (trial phase) and whether these improvements were sustained in the outcome retention phase, thus assessing the sustainability of our efforts.

Assessment letters across all three time periods consistently highlighted atypical sensory processing difficulties among young people diagnosed with movement disorders. Notably, eating challenges were frequently associated with sensory processing rather than rigidity or compulsions, with texture being cited as the primary reason for food rejection.^24^ Despite identifying these challenges, only a small proportion of letters during the trial period provided actionable recommendations or signposted resources to support sensory and eating behaviours. In contrast, during the outcome retention phase, while there was still only a small proportion of outcome letters signposting, these letters demonstrated much clearer signposting, offering specific support and advice for eating concerns, recommendations for further assistance and suggestions for reasonable adjustments at school.

Although co-occurring conditions such as autism, attention-deficit/hyperactivity disorder and obsessive-compulsive disorder are often screened for during initial assessments, challenges related to SEM are not routinely discussed. Several clinicians expressed surprise at the intensity and severity of eating issues and difficulties that emerged from a few individuals, once they inquired about SEM challenges. As a result of the project, the team liaised with the specialist feeding team within the service to access resources, including a standard information leaflet used with families about feeding issues. Additionally, the team is aiming to develop a leaflet relating to misophonia and its impact on mealtimes, as this issue arose during the project. While consultations with their onsite feeding service are available, if necessary, the team felt that the feeding issues seen in the clinic typically did not meet the threshold for referrals to this tertiary service. Instead, families could be signposted to other local community services for support or provided with recommendations during the assessment, which would then be summarised in the subsequent initial assessment letter. These adaptations are reflected in the continued identification of SEM challenges in outcome letters, with more tailored provisions evidenced in the outcome retention phase letters.

As a result of this project, we recommend that services working with neurodevelopmental movement disorder populations screen for SEM challenges during their assessments. There is a pressing need for more comprehensive and standardised assessment tools that capture the full spectrum of SEM challenges experienced by individuals with neurodevelopmental movement disorders. Ideally, brief self-report and parent-report screening tools that can be administered in waiting rooms would enable busy clinicians to promptly identify and address SEM experiences during assessments, considering the real-time constraints faced in clinical practice.

From a service user’s perspective, the lack of discussion on SEM could also reflect the predominant focus on the movements they identify as being most challenging. For example, tic-related eating and mealtime challenges may be overshadowed by other more problematic contexts or may not be considered severe enough to warrant discussion. Nonetheless, it is important to consider the broader impact of tics and stereotypies on mealtimes and quality of life. Research suggests that even when tics themselves do not directly interfere with eating or mealtimes, they can still act as barriers to mealtime activities and negatively affect quality of life due to associated challenges.^1 11^

Uncovering whether clinicians appropriately provided support for any of the challenges raised was a limitation of the current project. This would require knowing the nature and severity of the challenge and the clinician’s responses during the actual assessment. Reliance on letters as a proxy outcome measure may not have captured all the discussions that occurred during the assessments. The finding of more eating and mealtime concerns at baseline may reflect the lack of ability to capture these conversations, but also the baseline sample may simply have had a higher frequency of individuals with eating and mealtime concerns.

When considering the longer-term viability of the project, we note that during the retention phase (1 year after baseline and 3 months post-trial), SEM screening practices continued despite the absence of active reinforcement. This suggests a degree of sustainability. However, for SEM assessment to become an integral part of the service, key barriers must be addressed. This includes the need for a standardised manual or equivalent to guide the training and orientation of new staff on how to assess sensory, eating and mealtime concerns in individuals with tic disorders, especially considering the lack of awareness of the relevance of SEM for the movement disorder population. Additionally, a clear and consistent action plan is also needed to ensure that all clinicians follow a structured approach to managing identified eating concerns. Addressing these barriers will be essential to embedding SEM assessment into routine practice and ensuring long-term service sustainability.

To support long-term sustainability, future efforts will focus on embedding SEM screening more firmly within clinic infrastructure. This includes incorporating SEM prompts into standardised assessment forms and developing a brief clinical checklist to guide consistent inquiry during initial consultations. SEM assessment will also be included in staff induction and ongoing training, supported by the development of written guidance on referral thresholds and recommended responses. To maintain accountability, SEM documentation will be reviewed as part of routine audit cycles, with findings shared in team meetings and supervision spaces. These measures aim to support consistent, high-quality practice and enable the team to address any emerging gaps in a timely and systematic way.

Best practice would involve comprehensive documentation of challenges raised and support given (including psychoeducation), and, where possible, signposting to materials that support consolidation and adherence to any psychoeducation provided. In some cases, the severity of challenges may warrant a referral for more specialist support, highlighting the need for clinicians to understand relevant referral pathways for complex feeding and/or eating disorder services.

Future research and QI initiatives could support the development of accessible materials offering self-help guidance and advice. Qualitative research methodologies may be employed to investigate barriers and facilitators in implementing interventions addressing SEM challenges in clinical practice. Additionally, efforts could focus on developing and evaluating strategies to enhance the integration of SEM screening and management into routine clinical assessments for tics, ensuring that individuals receive comprehensive support tailored to their specific needs.

## Conclusion

The findings of this study suggest that incorporating routine screening for SEM challenges into neurodevelopmental movement disorder assessments can help identify unmet needs and highlight areas requiring additional support. Although integrating such practices into standard clinical procedures offers clear benefits, feasibility may be constrained by time limitations and competing clinical priorities. However, this study demonstrates that a multidisciplinary approach and collaboration among health professionals and services can provide a more comprehensive framework for managing the challenges faced by young people with neurodevelopmental movement disorders.

This project underscores the clinical importance of identifying SEM challenges and effectively addressing them in practice. Future research and QI initiatives are necessary to enhance the identification and management of SEM challenges. These should focus on developing population-specific resources, improving referral pathways and creating tools that support efficient, sustainable integration of SEM screening into routine clinical workflows. Embedding SEM assessment into clinic infrastructure through standardised forms, checklists, staff training and regular audits will be key to ensuring long-term impact and service transformation.

## Data Availability

No data are available.
